# Current News

**Published:** 1894-12

**Authors:** 


### Current News.

### WOMAN'S DENTAL ASSOCIATION OF THE UNITED STATES.

The regular monthly meeting of the Woman's Dental Association of the United States was held October 6, 1894, at Dr. Focht's office, in the Bank Building, Broad and Columbia Avenue, Philadelphia, Dr. Anna T. Focht, President, in the chair.

Subject of the evening, "The Care of Patients," by Dr. Annie Felton Reynolds, of Boston.

In the absence of the essayist, the paper was read by Dr. Mary H. Stilwell.

The next meeting will be held at Dr. Maria T. Lasser's office, 1602 Arch Street, November 3, 1894.

Emily W. Wyeth,
*Recording Secretary.*

3920 Fairmount Avenue.

---

### OHIO STATE DENTAL SOCIETY.

The Ohio State Dental Society will hold its next annual meeting at Neil House, Columbus, Ohio, December 4 to 6, 1894.

J. R. Callahan,
*Chairman Executive Committee.*

